# Expert knowledge-based peak current mode control of electrosurgical generators for improved output power regulation

**DOI:** 10.2478/joeb-2023-0005

**Published:** 2023-11-17

**Authors:** Muhammad Mohsin Rafiq, Asier Ibeas, Nasim Ullah

**Affiliations:** 1. Electrical Engineering, CECOS University of IT & Emerging Sciences, Peshawar, Pakistan; 2. Dept. of Telecomm. and Systems Eng., Faculty of Engineering, Universitat Autònoma de Barcelona, Barcelona, Spain; 3. Department of Electrical Engineering, College of Engineering, Taif University Saudi Arabia, Taif, Saudi Arabia, nasimullah@tu.edu.sa

**Keywords:** Electro-surgery, tissue burning, tissue impedance, Peak current mode control, Expert knowledge control

## Abstract

Electrosurgical generators (ESG) are widely used in medical procedures to cut and coagulate tissue. Accurate control of the output power is crucial for surgical success, but can be challenging to achieve. In this paper, a novel expert knowledge-based peak current mode controller (EK-PCMC) is proposed to regulate the output power of an ESG. The EK-PCMC leverages expert knowledge to adapt to changes in tissue impedance during surgical procedures. We compared the performance of the EK-PCMC with the classical peak current mode controller (PCMC) and fuzzy PID controller. The results demonstrate that the EK-PCMC significantly outperformed the PCMC, reducing the integral square error (ISE) and integral absolute error (IAE) by a factor of 3.88 and 4.86, respectively. In addition, the EK-PCMC outperformed the fuzzy PID controller in terms of transient response and steady-state performance. Our study highlights the effectiveness of the proposed EK-PCMC in improving the regulation of the output power of an ESG and improving surgical outcomes.

## Introduction

Electrosurgical generators (ESGs) are medical devices that are used to generate high-frequency electrical current for cutting and coagulating tissues during surgical procedures [1].

Electrosurgical devices have come a long way, focusing on enhancing precision and effectiveness in medical procedures. The journey began in the early 1900s, with a major breakthrough occurring in 1968 when solid-state generators were introduced, laying the foundation for future improvements. In the mid-1990s, electrosurgery generators started utilizing intelligent feedback systems known as tissue response or tissue effect generators, also referred to as instant response technology. These systems rapidly comprehend tissue behavior, leading to more precise surgeries.

In the late 2000s, a significant advancement emerged in electrosurgery through the Covidien Force Triad. This device could provide consistent power, although in certain cycles, power deviations reached as high as 120 W [6]. Additionally, the Force Triad incorporates PID control. A few years later, the Valleylab Force FX8 from Covidien came onto the scene. The FX8 managed to enhance performance five times compared to the Force Triad, as indicated by test reports from Medtronic’s. This improvement meant that the FX8’s power deviations could be as low as 24 W.

Nowadays, these generators typically operate at frequencies in the range of 300 kHz to 5 MHz [2], and they are able to deliver a variety of output power levels to meet various surgical requirements. Most commercially available ESGs operate at a frequency of 500 kHz ± 100 kHz. Increasing the frequency range to MHz amplifies the implications of frequency on safety, particularly the antenna effect and the risk of potential burns due to increased leakage currents. Consequently, there are low-power ESGs available for dental and plastic surgeries that operate at 4 MHz.

Furthermore, accurately controlling the output power of electrosurgical generators is still challenging, as the load characteristics of the tissue can vary significantly during the procedure [3].

**Figure 1:**
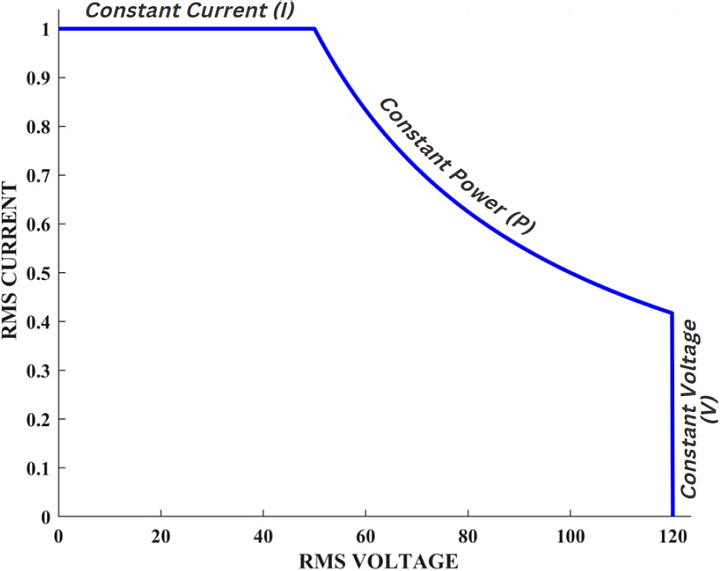
Desired output characteristic. Current in amperes and voltage in volts.

Inaccurate control of the output power of electrosurgical generators can lead to variations in the surgical outcomes, such as too much power can cause excessive tissue damage, while too little power can result in inadequate tissue treatment [4–6]. The electrical impedance of the circuit comprising the ESG and tissue varies significantly based on the type of tissue, and can typically range from ten of ohms to thousands of ohms [7]. To ensure a stable power output and account for the fundamental heating effect, it is ideal for the ESG output to be a constant power source. However, in practical terms, this constant power output needs to be restricted to a maximum voltage and current to ensure the safety of the ESG, electrosurgical instruments, and leads, all of which have a limited capacity to carry current and insulate against voltage. An I/V plot of the ESG’s ideal output characteristics is shown in Figure 1 [8].

To prevent the formation of arcs that can be hazardous during surgery, it is essential to regulate the maximum voltage output of the ESG. In high-impedance tissue sections, a significant amount of power can generate high voltages that trigger an arc between the electrosurgical instrument and the patient. These arcs reduce the surface area of the instrument by striking a smaller area, leading to higher current density in the tissue and a greater risk of carbonization. Therefore, regulating the maximum voltage output of the ESG is crucial in achieving the desired clinical outcomes. To achieve “constant power” in an AC output, it is necessary to ensure that the average power delivered during each switching cycle remains constant.

Electrosurgical generators offer various power settings for selection, which depend on different factors such as tissue type, surgical goal, surgeon experience, and preference. Although the manufacturer’s user manual or reference values may provide constant power output, the surgeon’s expertise and judgement are crucial in determining the appropriate value. Several references including [9–12] provide further information on the selection of constant power output in electrosurgical generators. The surgeon may adjust the constant power output value during surgery based on tissue response and other factors, necessitating a comprehensive understanding of the electro-surgical generator and its impact on tissue. The selection of constant power output values is a crucial element of electrosurgery, and the surgeon must carefully consider it to achieve the desired surgical outcome while minimizing tissue damage.

This paper introduces a novel approach that addresses a critical gap in electrosurgical generator technology — power deviation. Unlike existing control methods, our proposed controller ensures consistent outcomes and significantly reduces power deviation compared to the literature and state-of-the-art devices currently available in the market. This strategy’s innovation lies in its integration of expert knowledge into peak current mode control, effectively overcoming challenges posed by varying load impedance conditions. By segmenting the non-linear ideal power curve into distinct regions and employing a curve-fitting technique, the approach enhances accuracy and stability, resulting in more reliable and precise power output regulation. Through simulations, we validate the superior performance of our expert knowledge-based strategy when compared to conventional methods, offering a promising solution for improved power regulation across a range of applications.

The control of electrosurgical generators (ESGs) can be separated into two components: architecture and control technique. The architecture refers to the physical circuitry used to regulate the power output of an ESG. One approach that has been proposed in [13] for ESG that is capable of fast regulation of output power along with the advantage of generating arbitrary waveforms and current shapes. However, the method outlined in [13] produces square-wave output waveform with a significantly high switching frequency, resulting in more switching losses. More importantly, square waves generate super-harmonics which potentially damages tissues, or soft switching inverters, which lower switching loss but can only be beneficial for a small load range [3]. Furthermore, the load range is also significantly below those required to support electrosurgery [14] [15]. Therefore, the multi resonant-frequency (MRF) approach has been proposed in [16] to address the challenges faced by high-frequency inverters in the electrosurgery field. However, MRF and other common architectures are based on resonant inverters [17,18]. As a result of the resonant inverters’ self-limiting characteristics [19], the maximum output current and voltage have been set naturally. In addition, they create a near-sinusoidal waveform with very little output frequency flexibility, with the consequences that a high peak instantaneous power is produced for a specific average power. An alternative design for ESG that utilizes a buck-boost topology, is described in [20]. It operates as a square wave current source, which helps maintain the instantaneous power at a level closer to the desired average power.

Control technique, on the other hand, refers to the algorithm or strategy used to regulate the output power of an ESG. Several low-frequency control methods have been proposed, including PI control [21], PID control [22–24], adaptive PID control [25], fuzzy control [7,26,27], neural network control [28], and fractional-order PID control [29]. These control loops utilize measurements of power over multiple cycles to achieve the desired output power [30–32]. In [20], a peak current mode control approach was proposed to regulate the peak value of the inductor current in each cycle. However, this approach can result in non-ideal behaviors such as peak to average error (PAE) [33], artificial induced ramp and transient error, leading to excessive power delivery and inconsistent cutting speed and drag force. Compensation techniques have been proposed to reduce peak-to-average error [34,35], but they may not be effective when the converter operating point varies significantly.

To address the identified issues and improve the regulation of the output power based on the peak value of the current, this research paper proposes an expert knowledge-based peak current mode control (EK-PCMC) strategy. The novelty of this approach lies in its utilization of expert knowledge to enhance the performance of peak current mode control in constant power mode, particularly when dealing with varying load impedance. Traditionally, peak current mode control has faced challenges in accurately regulating power output under changing load impedance conditions. To overcome this, the proposed EK-PCMC strategy divides the non-linear ideal power curve into five distinct regions through careful analysis and consideration. Each region is then compensated using a curve-fitting technique that considers both the error signal and the output voltage. This approach results in precise estimation of the compensation value, allowing for improved accuracy and stability in power output regulation.

One of the key advantages of the EK-PCMC strategy is its adaptability and flexibility. It enables the incorporation of new knowledge and insights into the control strategy, making it well-suited for electrosurgical applications and other scenarios requiring precise current control with varying load impedance. The controller’s stability and robustness are enhanced, leading to more reliable and consistent power output regulation. This research paper’s main contribution is the integration of expert knowledge into the peak current mode control strategy, a novel approach not previously reported in the literature. To validate the effectiveness of the proposed EK-PCMC strategy, simulations were conducted, comparing its performance with existing control methods, including peak current mode control and fuzzy PID control. The simulation results demonstrate that the EK-PCMC strategy significantly reduces steady-state error compared to traditional peak current mode control. Moreover, the EK-PCMC strategy exhibits similar transient performance to peak current mode control. Additionally, when compared to fuzzy PID control and other existing control methods, the EK-PCMC strategy shows improved steady-state and transient performance. By leveraging expert knowledge to enhance peak current mode control, this research opens up new possibilities for improved power output regulation in various applications. The incorporation of expert knowledge allows for adaptability to changing conditions, which is essential in real-world scenarios. This research serves as a valuable contribution to the field of current control strategies and offers promising prospects for more reliable and efficient power regulation in diverse applications.

## Materials and methods

### Circuit diagram of electrosurgical generator and mathematical modeling of converters

**Figure 2:**
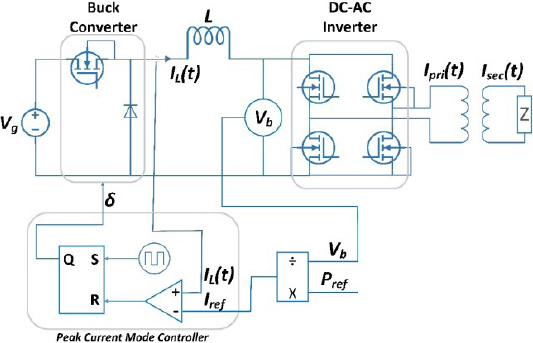
Circuit diagram of Electrosurgical generator.

The electrosurgical generator model consists of a DC-DC buck converter, a DC-AC inverter, and a control algorithm, as shown in Figure 2. The input to the system is *V_g_*, which serves as the power source, and it is used to generate the output voltage *V_b_*. The DC-DC buck converter steps down the input voltage to a lower voltage level, which is then supplied to the DC-AC inverter. The DC-AC inverter, in turn, converts the DC voltage to a high-frequency AC voltage that is used to drive the electrosurgical instrument.

*I_L_*(*t*) represents the inductor current, and *V_b_* is the buck converter output voltage and input voltage to high-frequency inverter. *I_pri_*(*t*) represents the isolated transformer primary current, while *I_sec_*(*t*) is the transformer secondary current that is delivered to the surgical instrument used by the surgeon.

In this diagram, the tissue impedance is represented by Z. Impedance is modeled as a resistor [20] to simulate and test the ESG.

### DC-DC buck converter

**Figure 3:**
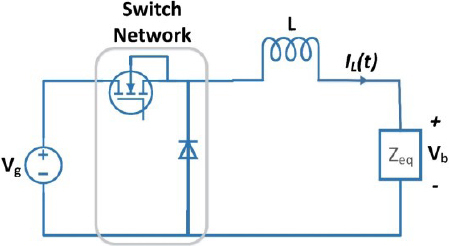
Buck converter.

The first stage of the circuit is a buck converter as shown in Figure 3, which achieves the reduction of the DC input voltage by controlling the duty cycle.

*Z_eq_* is equal to the reflected impedance of an ideal transformer connected with a DC to AC square wave inverter. The reflected impedance of an ideal transformer can be calculated using the turns ratio of the transformer [36]. From table 2, the transformer turn ratio *N* is 1/3. Therefore, *Z_eq_* can be calculated as *Z_eq_* = *Z* × *N*^2^, which gives *Z_eq_* = *Z* / 9.

The voltage drop across the inductor is determined by the rate of change of current passing through it, mathematically represented as $V_{L}=L\frac{dI}{dt}$, *L* symbolizes the self-inductance of the circuit, *V_L_* denotes the voltage across the inductor, and *I* represent the current flowing through it. In the context of a buck converter circuit, the value of the inductance is defined as per the following specifications:

1 $$L=\frac{V_{b}\times \left(V_{g}-V_{b}\right)}{\text{Δ}I\times f_{s}\times V_{g}}$$

In equation 1, (*V_b_*) is output voltage of buck converter, (*V_g_*) is input voltage and (*f_s_*) represents switching frequency. Using Table 1, the inductance at *f_s_* = 1000 kHz is determined to be 100 pH. The design specifications of the buck converter are tabulated in Table 1.

**Table 1:** Parameters of buck converter.

Parameter	Value	Unit
Input voltage (*V_g_*)	50	Volt
Maximum output voltage (*V_b_*)	45	Volt
Maximum average current (*I_avg_*)	3	Ampere
Duty cycle (D)	46	Percent
Switching frequency (*f_s_*)	1 x 10^6^	Hz
Inductance (*L*)	100 x 10^−6^	Henry

The parameters in table 1 are calculated based on the requirements of the final output of electrosurgical unit based on [20].

### High frequency DC-AC inverter

**Figure 4:**
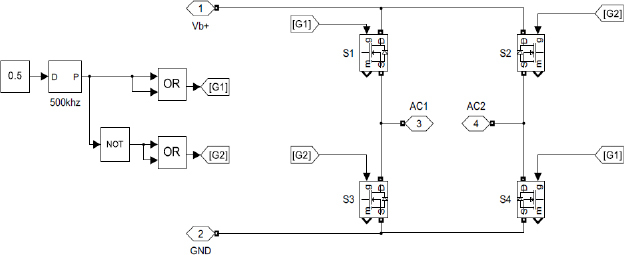
High frequency DC-AC Inverter.

As illustrated in Figure 2, the second stage of the circuit is the inverter unit. The high-frequency inverter, shown in Figure 4, is full bridge high frequency dc to ac inverter which is used to convert the DC current to high frequency AC current [37,38].

The operation of the full-bridge DC to AC square wave inverter can be explained mathematically by dividing the circuit into two halves - the upper half and the lower half [37]. Each half consists of two switches. During the first half of the operation, the upper left switch (S1) and the lower right switch (S4) are turned on, while the upper right switch (S2) and the lower left switch (S3) are turned off. This results in a voltage across the load of *V_ac_* = *V_b_*, where *V_b_* is the input DC voltage. This generates a positive half-cycle of the AC square wave.

During the second half of the operation, the upper right switch (S2) and the lower left switch (S3) are turned on, while the upper left switch (S1) and the lower right switch (S4) are turned off. This results in a voltage across the load of *V_ac_* = −*V_b_*, which generates a negative half-cycle of the AC square wave.

The output frequency of the full-bridge DC to AC square wave inverter is determined by the switching frequency of the switches. The duty cycle of the switches can be adjusted to control the output voltage and power [37]. but in this application inverter is operating at fixed duty cycle of 50%. the switching frequency of the inverter for electrosurgical applications is 500 kHz [7]

During the first half-cycle of the inverter operation:

2 $$V_{ac}=V_{b}$$

During the second half-cycle of the inverter operation:

3 $$V_{ac}=-V_{b}$$

The design specifications of this inverter circuit are presented in Table 2.

**Table 2:** Parameters of inverter.

Parameter	Value	Unit
Maximum Input DC voltage (*y_fc_*)	45	Volt
Maximum output voltage (*V_RMS_*)	120	Volt
Maximum output current (*I_RMS_*)	1	Ampere
Switching frequency (*f_s_*)	500 x 10^3^	Hz
Transformer turn ratio	1:3	-
Impedance range	10-340	Ohms
Reference power command	50	Watt

Electrosurgical output requirements and impedance range are based on [7,15]

### Ideal output curve for an electrosurgical generator and mathematical modeling

The ideal output curve for an electrosurgical generator consists of three modes: constant current, constant power, and constant voltage [20], as shown in Figure 1.

#### Constant current mode

**Figure 5:**
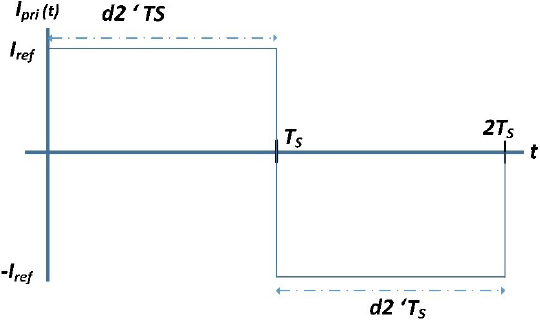
CMC buck output current waveform.

In constant current mode, the generator maintains a constant output current irrespective of the load impedance. It is a well-established fact that the buck converter output will exhibit a constant DC current source characteristic when controlled in current mode control (CMC) as reported in reference [39]. An example of a CMC controlled buck converter connected in series with a DC-AC inverter is shown in Figure 2. With the DC to AC inverter operating at a duty cycle of 50%, the output will be an AC current source of the same magnitude as illustrated in Figure 5. This results in the desired AC constant current source.

**Figure 6:**
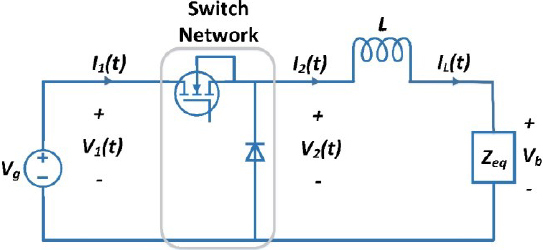
Switch network terminal quantities of buck converter.

**Figure 7:**
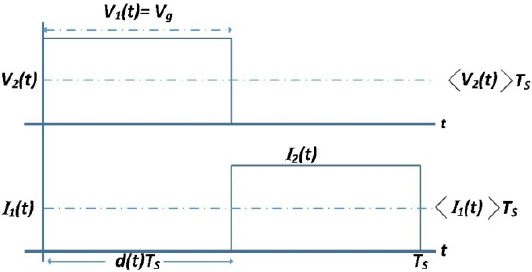
Waveforms for circuit.

The output of a CMC control buck converter can be represented as a continuous current source through the use of averaged switch modeling. Equation (4) presents the averaged equations for the switch network terminal values of the buck converter shown in Figures 6 and 7.

4 $$\begin{array}{ll} {〈I_{1}(t)〉}_{T_{s}} & =d(t)\cdot {〈I_{2}(t)〉}_{T_{S}}\\ {〈v_{2}(t)〉}_{T_{s}} & =d(t)\cdot {〈v_{1}(t)〉}_{T_{s}} \end{array}$$$$\matrix{
{{{\left\langle {{I_1}(t)} \right\rangle }_{{T_s}}}} \hfill & { = d(t) \cdot {{\left\langle {{I_2}(t)} \right\rangle }_{{T_S}}}} \hfill \cr
{{{\left\langle {{v_2}(t)} \right\rangle }_{{T_s}}}} \hfill & { = d(t) \cdot {{\left\langle {{v_1}(t)} \right\rangle }_{{T_s}}}} \hfill \cr
} $$

The equations state that the time-averaged value of the input current, denoted as 〈*I*_1_ (*t*)〉*_T_s__*, can be calculated by the product of the time-averaged value of the output current, 〈I_2_ (*t*)〉*_T_s__*, by the duty cycle, *d*(*t*). This indicates that the inductor current depends on the ratio of the output current and the duty cycle.

The equation (4) represents the relationship between the time-averaged value of the output voltage of the buck converter, 〈*𝑣*_2_ (*t*)〉*_T_s__*, and the duty cycle, *d*(*t*), and the time-averaged value of the input voltage to the buck converter, 〈*𝑣*_1_ (*t*)〉*_T_s__*. It states that the output voltage is obtained by multiplying the duty cycle with the input voltage. This highlights the influence of the duty cycle on the output voltage of the buck converter.

If the CMC controller is able to maintain the average inductor current within the control current limit, then:

5 $${〈i_{2}(t)〉}_{T_{s}}={〈I_{L}(t)〉}_{T_{S}}={〈I_{\text{ref }}(t)〉}_{T_{S}}$$

Eq (6) can be obtained by eliminating the duty cycle from (4):

6 $$\begin{array}{ll} d(t) & =\frac{{〈v_{2}(t)〉}_{T_{S}}}{{〈v_{1}(t)〉}_{T_{S}}}\\ {〈I_{1}(t)〉}_{T_{S}} & =\frac{{〈v_{2}(t)〉}_{T_{S}}}{{〈v_{1}(t)〉}_{T_{S}}}\cdot {〈I_{2}(t)〉}_{T_{S}} \end{array}$$

Eq (7) can be written by substituting the value of 〈*I*_1_ (*t*)〉*_T_s__*from (5).

7 $${〈I_{\text{ref }}(t)〉}_{T_{s}}\cdot {〈v_{2}(t)〉}_{T_{s}}={〈P(t)〉}_{T_{s}}={〈I_{1}(t)〉}_{T_{s}}\cdot {〈v_{1}(t)〉}_{T_{s}}$$

**Figure 8:**
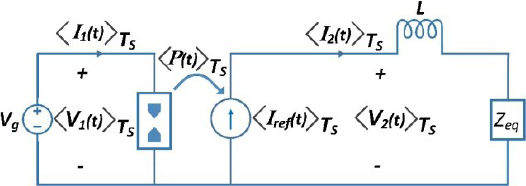
Averaged switch network derived from equation (7).

The behavior of a buck converter controlled by peak current mode (PCM) is described in Equation (7). The converter’s input port is designed to consume a constant amount of power 〈*P* (*t*)〉*_T_s__* and the PCM controller adjusts the duty cycle to ensure that the voltage output 〈*𝑣*_2_ (*t*)〉*_T_s__* varies in order to supply all the power input to the load for a fixed value of 〈I_ref_ (*t*)〉*_T_s__*. The switch network is illustrated in Figure 8.

The model shown in Figure (8) represents a simplified version of a PCM controller buck converter with constant output current source.

#### Constant Power Mode

In constant power mode, the generator maintains a constant output power irrespective of the load impedance. In the constant power mode, the power input to the inverter switch network is represented by equation (8).

8 $$P=I_{L}(t)\cdot \delta \cdot V_{g}$$

As we know that in PCMC inductor current peak is equal to *I_ref_* then equation (8) becomes:

9 $$P=I_{\text{ref }}\cdot \delta \cdot V_{g}$$

At a 50% duty cycle, the DC-AC inverter inverts the voltage without altering the amplitude of the DC-DC buck voltage. As a result, *I_pri_*(*t*) = *I_L_*(*t*) = *I_ref_*. Equation (9) consists of two variables that change over time (*I_ref_* and *δ*) and can be used to calculate the output power P for a fixed load. This equation can be implemented by using the duty cycle expression in (10), where 0 ≤ *t* ≤ *T_s_*.

10 $$\delta =\frac{t}{T_{s}}$$

The value of *I_ref_* can be calculated by substituting the duty cycle expression from equation (10) into equation (9). This results in the equation presented in (11):

11 $$\begin{array}{ll} P & =I_{ref}\cdot \frac{t}{T_{s}}\cdot V_{g}\\ I_{\text{ref }} & =\frac{P}{V_{g}}\cdot \frac{T_{s}}{t} \end{array}$$

Equation (11) defines the average inductor current, denoted as *I_ref_*. The constant *P* represents the reference power set by the user, while *V_g_* remains constant as the input voltage to the buck converter. Moreover, the ratio $\frac{T_{S}}{t}$ is equal to $\frac{1}{d}$,where *d* represents the duty cycle.

#### Constant Voltage Mode

**Figure 9:**
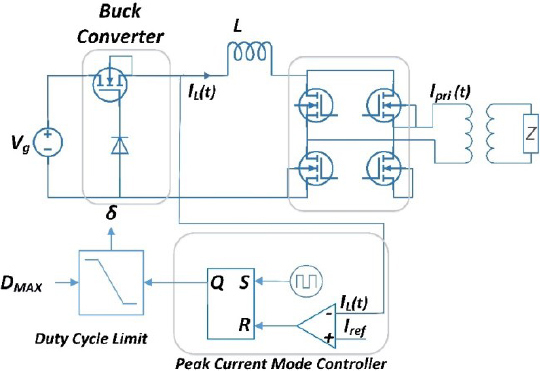
Maximum voltage limit using duty cycle.

To achieve a constant output voltage, a better approach is to limit the duty cycle of the existing PCM controller, as depicted in Figure 9.

It is important to acknowledge that using duty cycle limiting to achieve a constant voltage output on a PCM controller may come with some drawbacks such as reduced efficiency and increased output voltage ripple. By placing a restriction on the duty cycle of the peak current mode controller (PCM) in a DC-DC converter, it operates in an open-loop mode that may lead to instabilities.

In conclusion, this section provides the circuit diagram of an electrosurgical generator, including a DC-DC buck converter and a DC-AC inverter, and the mathematical modeling of these converters. Additionally, we presented the ideal output curve for an electrosurgical generator, including constant current, constant power, and constant voltage, with mathematical modeling for each mode.

### Base Control and limitations

In the previous section, we discussed the circuit diagram and mathematical modeling of an electrosurgical generator, as well as the ideal output curve for constant current, constant power, and constant voltage modes. In this section, we will explore the base control method for regulating the output current of the electrosurgical generator. This technique is also known as peak current mode control. We will discuss the advantages and limitations of this control method and analyze its impact on the electrosurgical generator’s performance.

### Base control (peak current mode control)

Base control is peak current mode (PCM) control. The Peak Current Mode (PCM) control is a technique used to regulate a specific current in a converter. It is used to regulate the inductor current by measuring it and comparing it to a reference value, *I_ref_*. The control strategy involves determining the switching waveform, δ, based on this comparison.

**Figure 10:**
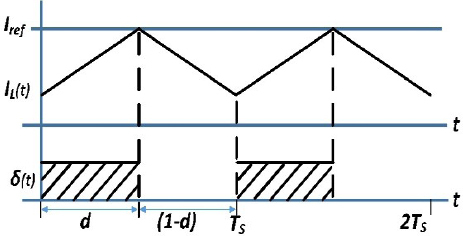
Waveforms in PCM control.

**Figure 11:**
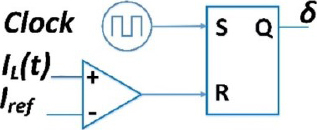
Realization of Peak current mode controller.

As shown in the waveforms of Figure 10, when the ripple of the inductor current is minimal, the average of the inductor current is closely matched with the current control limit. However, as the ripple amplitude increases, the difference between the average of current *I_L_* and the control current limit *I_ref_* also increases. A straightforward approach to implementing a PCM controller is depicted in Figure 11.

**Figure 12:**
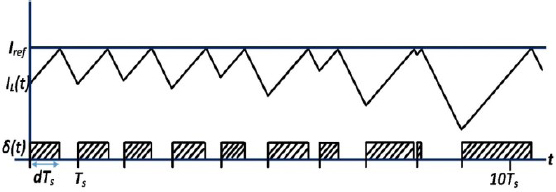
PCM control waveforms showing instability

In Figure 11, a set-reset (S/R) latch is used to generate the drive signal δ. The input is supplied by the periodic clock to the set pin, which initiates the interval d. A comparator is used to monitor the inductor current and to reset the latch when the current in inductor surpasses the control limit; it triggers the end of the d interval. However, it is worth noting that PCM control may become unstable for duty cycles greater than 50%. Figure 12 illustrates this instability by showing that when trying to function at duty cycles that are higher than 50%, the geometry of the inductor current waveform results in the circuit’s inability to achieve a steady operating point.

**Figure 13:**
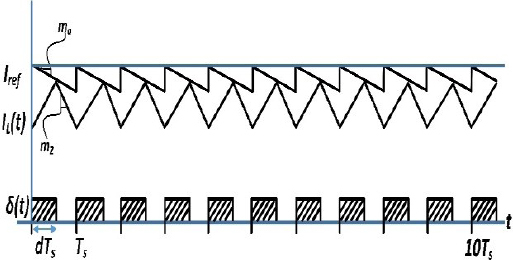
PCM controller’s stability is improved by adding an artificial ramp.

To ensure stable operation of the converter at duty cycles above 50%, one solution is to add an artificial ramp for compensation to the sensed inductor current or subtract it from the control current limit. This is illustrated in Figure 13. The addition of the artificial ramp creates a stable operating point, allowing the converter to operate at higher duty cycles [33].

The introduction of an artificial ramp in the Peak Current Mode controller, as depicted in Figure 13, demonstrates a notable improvement in stability when compared to the absence of an artificial ramp, as illustrated in Figure 12.

The minimum slope of this artificial ramp required for stability is dependent on the slope of the inductor current during the d interval. Equation (12) illustrates this relationship and can be used to determine the appropriate slope of the artificial ramp needed to ensure the stability of the converter.

12 $$m_{a,\min}=\frac{1}{2}m_{2}$$

As per reference [33], we can write the following equation for *m*_2_ :

13 $$m_{2}=\frac{V_{b}}{L}$$

Using equation (13), we can express equation 12 in the following form:

14 $$m_{a,\min}=\frac{V_{b}}{2\times L}$$

Therefore, equation (14) gives us the minimum value of slope of artificial ramp based on the given conditions.

### Limitation of Base control (PCMC)

The peak current mode control (PCMC) technique is widely used to regulate the maximum inductor current value in power converters. However, in real-world power sources, three main types of non-idealities can occur: peak to average error (PAE), artificial ramp error, and transient error.

#### Peak to Average Error (PAE)

Peak to Average Error (PAE) refers to the error that may occur between the average and maximum inductor current values when there is a significant inductor current ripple [33]. Various compensation strategies can be implemented to lower or eliminate PAE in certain PCM control applications [35,39]. However, these techniques may not be effective when the converter operating point varies significantly. Large inductor current ripple invalidates the formula for determining the current control limit, which is used to minimize inductor current ripple and set the steady-state inductor current to the necessary level for maintaining constant power output across a range of load impedances [20]. The difference between the control current limit and the average current in the inductor can lead to a maximum steady-state error in output power [40].

**Figure 14:**
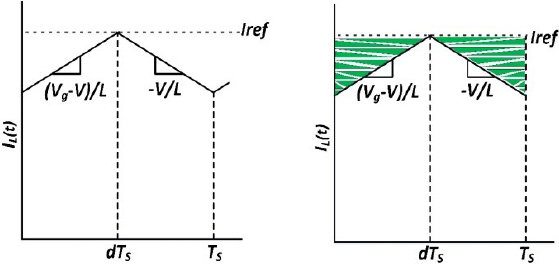
Steady-state inductor current.

Large inductor current ripple invalidates the formula 〈*I_L_* (*t*)〉*_T_s__* = *I_ref_* because, as shown in Figure 14, the peak inductor current value and the average inductor current value are not always equivalent.

**Figure 15:**
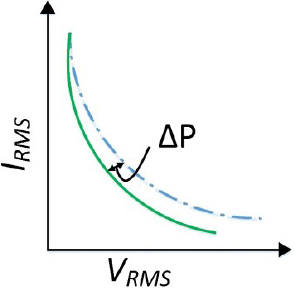
Peak to average error in ideal curve using PCMC.

The illustration in Figure 15 demonstrates how variations between peak and average values affect the steady output of a series buck inverter.

#### Artificial Ramp Error

**Figure 16:**
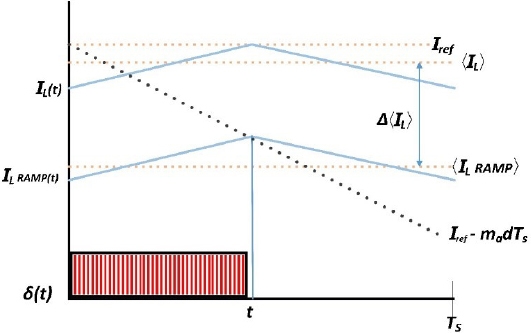
PCM control with and without artificial ramp.

Introducing an artificial ramp for compensation to the PCM controller can alter the PAE. The use of an artificial compensation ramp is known to improve noise immunity in the detected inductor current and enhance stability in PCM converters operating at a duty cycle of D = 50% or higher [39]. As depicted in Figure 16, this artificial compensation ramp effectively causes variation in the value of *I_ref_* that is dependent on the duty cycle.

It is important to note that the size of the artificial compensation ramp can affect the variation in the average current values of *I_L_*(*t*).

**Figure 17:**
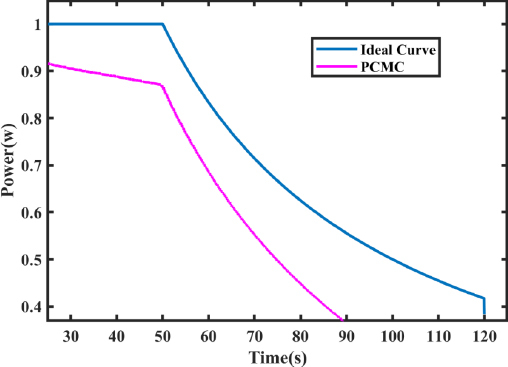
Impact of PAE and artificial ramp error.

The impact of this error on a constant power source is shown in Figure 17. It can be observed that there is a slight artificial ramp-induced error when the output impedance is low, but the error increases as the output impedance increases.

#### Transient Error

**Figure 18:**
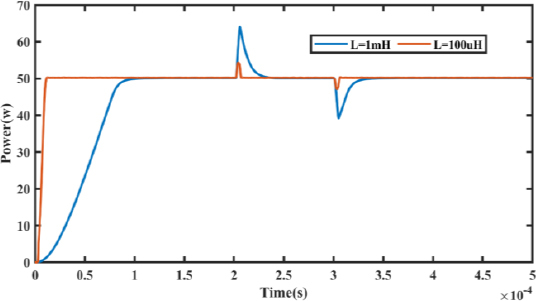
Transient response under step load.

Figure 18 illustrates a load step event at 0.2 milliseconds, resulting in a power overshoot that is more significant for higher values of *I_L_*. To optimize the performance of the PCM buck converter during transient conditions, it is essential to ensure that the stored energy in the inductor is discharged entirely into the load. Therefore, reducing the value of the inductor can be an effective approach to improve the performance of the converter.

In conclusion, these non-idealities can limit the performance of PCM control in power converters. However, various compensation strategies and optimization techniques can be applied to mitigate these limitations and improve the overall performance of the converter.

### Expert knowledge-based control

This section presents the design of an expert knowledge-based peak current mode controller. The proposed controller aims to regulate the current in a load with varying impedance to maintain a constant power output.

The analysis of peak current mode control in the previous section highlights three main factors that affect control accuracy: artificial ramp-induced error, peak to average error (PAE), and transient error. However, as previously mentioned, transient error is influenced by the inductor value of the buck converter and is a trade-off between transient response and ripple current. The remaining two factors can cause errors to increase with an increase in voltage, which corresponds to an increase in load impedance in constant power mode. However, it is important to note that the average current in peak current mode control, expressed in Equation (15) as 〈*I_L_* (*t*)〉*_T_s__*, may not be equal to the reference current *I_ref_*:

15 $${〈I_{L}(t)〉}_{T_{s}}\neq I_{\text{ref }}$$

The steady-state error presents a critical challenge in peak current mode control, resulting in undesired power deviations. The steady-state error is due to artificial ramp-induced error, peak to average error (PAE). Recognizing the significance of mitigating this error, our research focuses on developing an effective compensation strategy.

Initially, we explored applying a constant compensation value equal to the steady-state error to counter its impact. However, this simplistic approach proved effective only at a single point, failing to address error variations across a wide range of impedance values.

To comprehensively analyze the error behavior, we calculated the complete error curve for a conventional peak current mode controller. This involved a meticulous comparison of the reference current with the measured current while operating in constant power mode. Our subsequent efforts focused on developing an interpolation technique to derive an equation that accurately reflects the error based on voltage measurements. In constant power mode, where impedance varies and directly affects voltage and current, the relationship between reference current *I_ref_* and voltage *V_b_* is $I_{\text{ref }}=\frac{P}{V_{b}}$.

However, when we directly applied single equations derived from the interpolation to the controller, we observed inaccuracies in following the reference current and, consequently, the ideal power curve. To address this, we implemented a region-based approach by dividing the non-linear ideal curve into multiple regions.

In order to strike a balance between accuracy and computational efficiency, we chose to divide the non-linear ideal curve into five regions, each with unique characteristics. This decision was made after conducting extensive experiments, progressively increasing the number of divided regions to 4 and 5. As expected, the error in current and power decreased as we increased the number of regions. However, upon reaching 6 regions, we observed minimal error impact, indicating that further increasing the number of regions would be impractical due to increased system cost.

**Figure 19:**
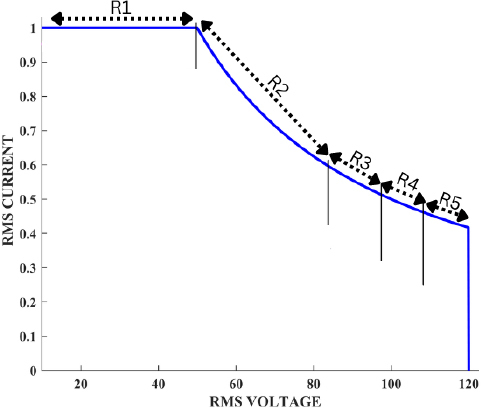
Rules derivation from ideal curve.

Thus, we determined that dividing the non-linear ideal curve into 5 regions as shown in Figure 19 offered the best balance between accuracy and computational efficiency. Each region was characterized by unique attributes, and we derived specific compensation equations for each segment using a curve-fitting technique. This approach enables our controller to accurately follow the ideal power curve and effectively mitigate steady-state error across varying impedance values.

To derive the compensation equation for each region, a curve-fitting technique that takes into account the unique characteristics of each region is used. The resulting equation is then used to calculate the compensation value for that specific region, which is subsequently applied to the reference current. This process of applying compensation separately for each region enables the controller to effectively account for variations in load characteristics, resulting in more accurate and stable control. The average current in each load region is given by a general equation (16):

16 $${〈I_{L}(t)〉}_{T_{s}}=I_{\text{ref }}+\text{Δ}I_{\text{ref }}$$

where Δ*I_ref_* is the compensation value.

A curve-fitting technique is used to derive the compensation equation for each region, which takes into account the error signal and the output voltage. The resulting equation is then used to calculate the compensation value for that specific region, which is subsequently applied to the reference current.

The compensation equations for 5 regions are given by:

Region 1: Rule: If *V_b_* < 17v, then compensation is

Δ*I_ref_* = 0.703595 × *log*(3.10892 × *V_b_* + 82.9714)

− 3.0551

Region 2: Rule: If 28 > *V_b_* ≤ 17v, then compensation is

Δ*I_ref_* = 0.703595 × *log*(3.10892 × *V_b_* + 82.9714)

− 3.048 − 0.0005 × *V_b_*

Region 3: Rule: If 32 > *V_b_* ≤ 28v, then compensation is

Δ*I_ref_* = 0.703595 × *log*(3.10892 × *V_b_* + 82.9714)

− 3.048 − 0.04/*log*(*V_b_*)

Region 4: Rule 1: If 36 > *V_b_* ≤ 32v, then compensation is

Δ*I_ref_* = 0.703595 × *log*(3.10892 × *V_b_* + 82.9714)

− 3.048 − 0.02/*log*(*V_b_*)

Region 5: Rule: If *V_b_* ≥ 36v, then compensation is

Δ*I_ref_* = 0.703595 × *log*(3.10892 × *V_b_* + 82.9714)))

− 3.118 + 0.002 × *V_b_*

**Figure 20:**
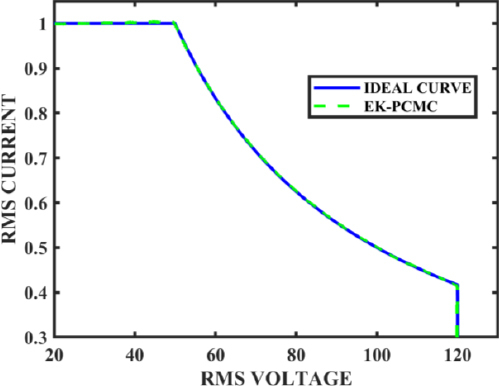
Output curve using EK-PCMC.

**Figure 21:**
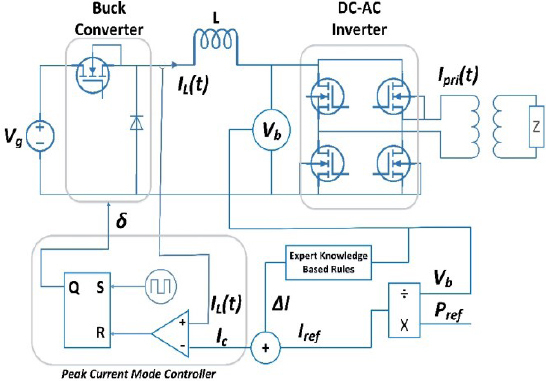
Block diagram of ESU with EK-PCMC control.

After implementing the compensation rules outlined above, the output power curve is depicted in Figure 20, and the block diagram of the EK-PCM control system is illustrated in Figure 21.

In Figure 20, two curves are presented: an ideal I-V curve and a curve obtained using EK-PCMC. The ideal curve corresponds to the curve featured in Figure 1, which serves as the standard curve with the specifications of 50 W constant power, 1A RMS current, and 120V RMS voltage.

In summary, the expert knowledge-based peak current mode control is a controller designed to regulate the current in a load with varying impedance to maintain a constant power output. The controller utilizes expert knowledge to optimize the performance of peak current mode control in constant power mode. Through analysis and curve fitting, we determine the compensation equations for the reference current based on the output voltage. By splitting the load range into five regions and applying compensation to the reference current separately for each region, we improve the controller’s stability and robustness. The proposed controller has the potential to enhance the performance of electrosurgical applications and can be applied to other applications that require precise current control with varying load impedance.

### Ethical approval

The conducted research is not related to either human or animal use.

## Results and Discussion

The purpose of this section is to demonstrate how incorporating an expert system can improve classical control methods. To achieve this objective, simulation studies were conducted using a mathematical model of an electrosurgical generator and a tissue load. The electrosurgical generator was modeled as a single-phase inverter connected to a buck converter, and the tissue load was represented as a resistor. Two sets of simulations were conducted to compare the performance of PCMC control with and without expert knowledge. One set of simulations used EK-PCMC control, while the other set utilized classical PCM control.

The aim of the simulations was to illustrate how integrating an expert system can enhance classical control methods compared to not using expert systems. Therefore, it is important to emphasize that the primary objective of these simulations is to showcase the efficacy of EK-PCMC control, in contrast to classical PCM control that does not incorporate expert knowledge.

**Figure 22:**
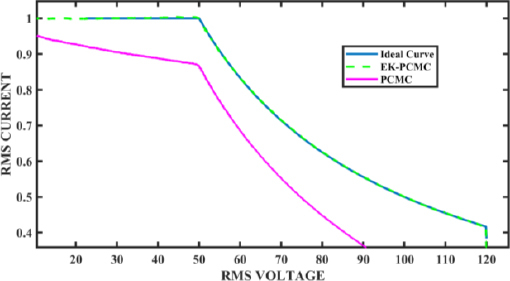
Comparison of VI curves for Ideal, PCMC, and EK-PCMC.

Figure 22 presents the VI curve of the ESG under a wide range of load variations, comparing the performance of the system with and without EK-PCMC control. The load variation was initiated at 340 Ω and linearly decreased to 10 Ω. To perform this simulation, a selected set of load values within the specified range were used, and the corresponding output power was measured at each point.

The simulation procedure involved setting the load resistance to a predefined value within the impedance range (e.g., 340 Ω) and recording the corresponding output power while using EK-PCMC control in the Simulink model. The same process was then repeated for various other load values within the specified impedance range until reaching the minimum load of 10 Ω. This allowed us to observe the system’s response to different load conditions in the simulated environment.

Likewise, the same procedure was carried out for the output power using traditional PCMC control in the Simulink model, serving as a comparison to the EK-PCMC control. For each load value, the output power was recorded, and the data were used to create a curve representing the relationship between load variation and output power in the simulated environment.

The resulting VI curve depicted in Figure 22 represents an aggregation of the data obtained for the selected load values within the entire impedance range. Each point on the curve corresponds to a specific load value and its respective output power measured during the simulation.

The key observation from the VI curve is that the EK-PCMC control in the Simulink simulation successfully maintains the ideal output power reference throughout the entire range of load variation. However, in contrast, the output power obtained using traditional PCMC control in the Simulink simulation displayed steady-state errors and artificially induced ramp errors as the load varied.

**Figure 23:**
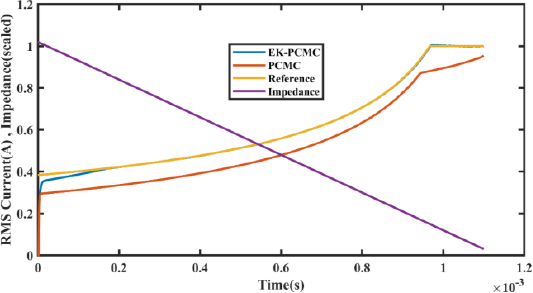
Output current comparison under load variations.

Figure 23 shows the RMS current of the ESG unit under the same simulations performed for Figure 22. In Figure 22, we presented the VI curve by measuring the voltage and current and plotting the data to demonstrate the system’s performance with and without EK-PCMC control. Similarly, in Figure 23, we focus solely on the current and plot it on the time axis to showcase the variations in current for both EK-PCMC and traditional PCMC control.

To enhance comparability with the current curve, we scaled down the impedance curve by a factor of 0.003. The scaling factor of impedance was calculated by dividing the maximum value of the current curve by the maximum value of impedance, allowing for a more meaningful representation of the current behavior.

While Figure 22 provides an overall view of the system’s response to load variations in VI curve form, Figure 23 allows us to specifically examine the behavior of the current over time. By doing so, we can gain insights into the dynamic nature of the ESG unit’s current when subjected to different load conditions.

Upon analyzing both figures, it becomes evident that the RMS current with EK-PCMC control consistently surpasses the current obtained with traditional PCMC control at all points of load variation. This observation further reinforces the effectiveness of the EK-PCMC control in maintaining a more stable and robust current output across varying load conditions.

**Figure 24:**
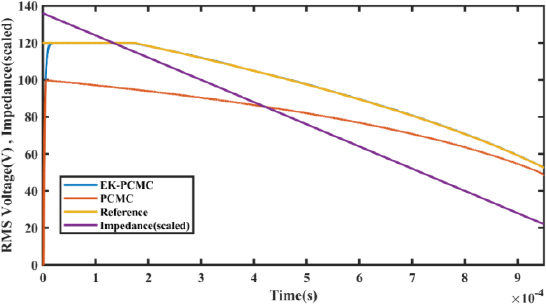
Output voltage comparison under load variations.

Figure 24 shows the RMS voltage of the ESG unit under a wide range of load variations. Like for Figure 23, we conducted simulations as described earlier for Figure 22, but here our focus is solely on the voltage behavior.

To ensure better comparability with the voltage curve, we scaled down the impedance curve by a factor of 0.4. This scaling factor was determined by dividing the maximum value of the voltage by the maximum value of impedance.

The figures clearly illustrate that the RMS voltage with EK-PCMC control consistently surpasses the voltage obtained with the traditional PCMC controller at all points of load variation. This observation underscores the effectiveness of the EK-PCMC control in maintaining a higher and more stable voltage output throughout the entire range of load variations.

We now present the results of our simulations comparing the performance of our proposed controller EK-PCMC, with a reference paper [7] on fuzzy PID control. The simulations were carried out for a reference power of 50 W, under two scenarios: (1) decreasing load steps, and (2) increasing load steps.

For scenario (1), the simulation was conducted with the same parameters as those presented in the reference paper [7]. The impedance values were set to 90 Ω for the first layer, 60 Ω for the second layer, and 30 Ω for the third layer, which changed at times 0, 0.001, and 0.002, respectively. To facilitate comparison with the power curve, the impedance curve was scaled down by a factor of 0.2. Our proposed EK-PCMC controller was able to adjust for the dip in power that occurred when the impedance value decreased.

**Figure 26:**
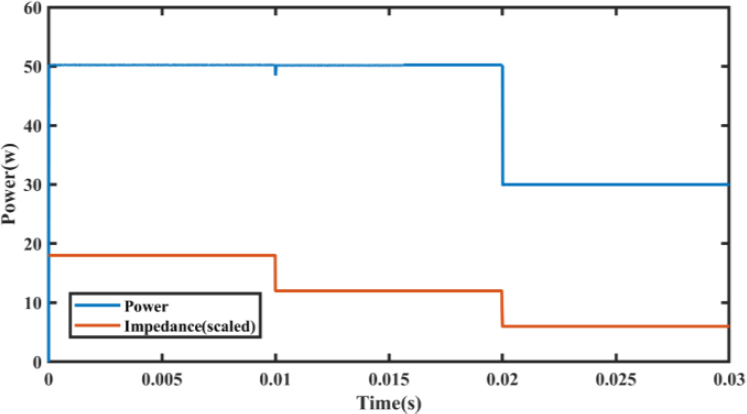
Output Power for decreasing load steps.

**Figure 27:**
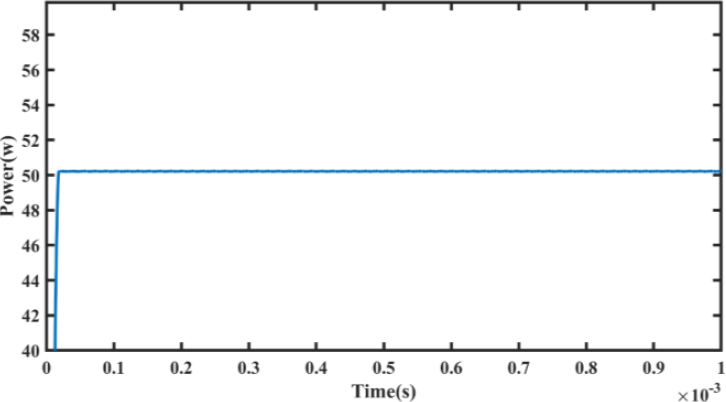
Output power (zoomed).

Furthermore, in the third layer, where the impedance was set to 30 Ω, the controller operated in a constant current mode to maintain a current limit of 1A. The simulation results are presented in Figure 26, with a zoomed-in view in Figure 27.

For scenario (2), the simulation was also carried out using the same parameters as those presented in the reference paper [7], with the impedance values set to 90 Ω for the first layer, 120 Ω for the second layer, and 150 Ω for the third layer, which changed at times 0, 0.001, and 0.002, respectively.

**Figure 28:**
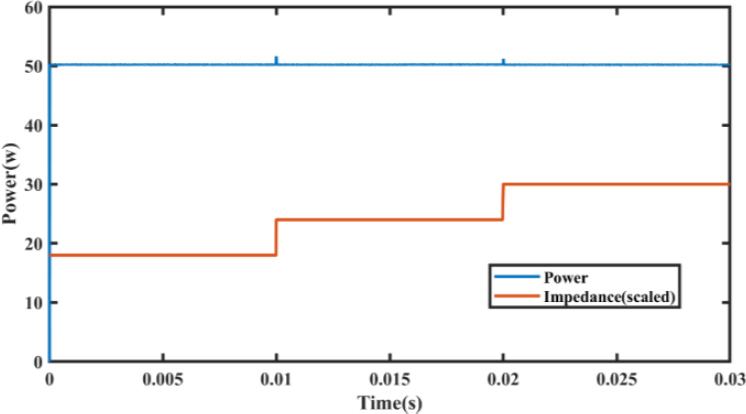
Output Power for increasing load steps.

Like for scenario (1), the impedance curve was scaled down by a factor of 0.2 to facilitate comparison with the power curve. Our proposed EK-PCMC controller was able to adjust for the rise in power that occurred when the impedance value increased. The simulation results are presented in Figure 28.

In both scenarios, the EK-PCMC controller demonstrated its ability to adjust for changes in impedance and maintain a stable power output. The simulation results support the effectiveness of our proposed controller in regulating power output under varying load conditions when compared to the reference fuzzy PID control.

The presented results indicate that classical peak current mode control (PCMC) exhibits a steady-state error in the power curve, which is not ideal for electrosurgical applications where precision and accuracy are essential. In contrast, the proposed controller minimizes the steady-state error in the power curve, improving the overall performance of the system.

Furthermore, the presented results highlight that the power curve exhibits large overshoot, long settling time, and large rise time with fuzzy PID and other classical controllers. In contrast, the proposed controller minimizes all these undesirable characteristics, by reducing the large rise time, settling time, and overshoot.

These improvements are critical for electrosurgical applications where precision and accuracy are essential. By minimizing the rise time, settling time, and overshoot, the proposed controller ensures that the output power quickly reaches the desired level and remains stable, eliminating potential damage to the surrounding tissue and improving surgical outcomes.

The aim of this study was to design and evaluate the performance of an expert knowledge-based peak current mode controller (EK-PCMC) for an electrosurgical generator. We compared the performance of the EK-PCMC with that of the traditional peak current mode controller (PCMC) and a fuzzy PID controller reported in a previous study. Our findings demonstrate that the EK-PCMC significantly outperforms both the PCMC and the fuzzy PID controller in controlling an electrosurgical generator.

Figure 22 demonstrates that utilizing EK-PCMC control enhances the generator’s capability to sustain a stable output power, unlike the variations observed in the output power when utilizing PCMC control, which is caused by the artificial ramp error and peak to average (PAE).

Furthermore, Figures 23 and 24 illustrate that the electrosurgical generator using EK-PCMC control exhibits an improved ability to sustain a stable output current and voltage across a wider range of loads.

Figure 25 presents a comparison of the reference power to the power obtained using EK-PCMC and PCMC control, with the impedance also being depicted to further highlight the effect of varying impedance levels. As depicted in the figure, the impedance decreases from 340 ohms to 10 ohms over the course of the measurement. At the start of the measurement, when the impedance is higher, the electrosurgical generator is operating in constant voltage mode. As the impedance decreases, at 17 milliseconds the generator transits to constant power mode and at 97 milliseconds, when impedance reaches 50 ohms, the generator shifts to constant current mode.

Table 3 shows that the EK-PCMC is approximately four times better than the traditional PCMC in reducing power error. This indicates that the use of expert knowledge in designing controllers can lead to a significant improvement in performance in controlling electrosurgical generators. Moreover, in comparison to the fuzzy PID controller reported in a previous study, the EK-PCMC controller exhibits better transient and steady-state performance, as shown in Table 4.

**Table 3:** Performance comparison between proposed and PCMC.

Controller	Integral square error (ISE)	Integral absolute error (IAE)
PCMC	0.3190	0.0175
EK-PCMC	0.0822	0.0036

**Table 4:** Performance comparison between proposed and previous work for increasing load.

Performance criteria	Previous work	EK-PCMC
*Step Response at 50W power at Impedance=90 ohm at time 0s*		
Power overshoot	4.20%	0.188%
Rise time of Power	0.89 ms	0.0047 ms
Settling time of power	2.1 ms	0.017 ms
*Load step from Z1 to Z2(90 ohm to 120 ohm) at time 0.01s*		
Power overshoot	9%	3.2%
*Load step from Z2 to Z3(120 ohm to 150 ohm) at time 0.02s*		
Power overshoot	10%	2.6%

Specifically, during a step response at 50 W power at an impedance of 90 ohms, the EK-PCMC exhibits a significantly lower power overshoot, shorter rise time, and shorter settling time than the fuzzy PID controller. Similarly, during load step changes from Z1 to Z2 and from Z2 to Z3, the EK-PCMC demonstrates significantly lower power overshoot than the fuzzy PID controller.

The proposed EK-PCMC control was also compared with a previous fuzzy PID controller for decreasing load impedances in Table 5. The EK-PCMC controller demonstrated significant improvements in power regulation for decreasing load impedances as well. The EK-PCMC control exhibited a power undershoot of 3.2% during the load step from Z1 to Z2, which was not applicable to the previous work. The rise time and settling time of power were also improved with the EK-PCMC controller as compared to the previous work.

**Table 5:** Performance comparison between proposed and previous work for decreasing load.

Performance criteria	Previous work	EK-PCMC
*Step Response at 50W power at Impedance=90 ohm*		
Power overshoot	4.20%	0.188%
Rise time of Power	0.89 ms	0.0047 ms
Settling time of power	2.1 ms	0.017 ms
*Load step from Z1 to Z2(90 ohm to 60 ohm) at time 0.01s*		
Power Undershoot	NA	3.2%
*Load step from Z2 to Z3(120 ohm to 30 ohm) at time 0.02s*		
Power Undershoot	NA	(In constant current mode)

## Conclusion

In conclusion, this research study presents a novel expert knowledge-based peak current mode controller (EK-PCMC) for regulating the output power of electrosurgical generators (ESG). The EK-PCMC leverages expert knowledge to adapt to changes in tissue impedance during surgical procedures, resulting in significantly improved performance compared to the traditional peak current mode controller (PCMC) and fuzzy PID controller.

Our findings demonstrate that the EK-PCMC outperformed the PCMC, reducing the integral square error (ISE) and integral absolute error (IAE) by a factor of 3.88 and 4.86, respectively. Moreover, the EK-PCMC surpassed the fuzzy PID controller in terms of transient response and steady-state performance. These results highlight the effectiveness of the proposed EK-PCMC controller in enhancing the regulation of output power in electrosurgical procedures.

The contributions of this study are twofold. Firstly, we introduce the concept of leveraging expert knowledge in the design of the EK-PCMC controller, which enables adaptive control based on tissue impedance changes. This innovative approach addresses the challenges associated with accurately controlling ESG output power and presents a significant advancement in the field.

Secondly, our research provides evidence of the superior performance of the EK-PCMC controller compared to existing control methods. The substantial reduction in power error, along with improved transient response and steady-state performance, showcases the potential impact of the EK-PCMC controller in enhancing surgical outcomes and patient safety.

Overall, the findings of this study highlight the significance of incorporating expert knowledge-based approaches in the design of control systems for ESGs. The proposed EK-PCMC controller offers a promising solution to improve the regulation of output power, ultimately contributing to more precise and efficient electrosurgical procedures. The outcomes of this research pave the way for further advancements in the field and underscore the importance of innovative control techniques in medical device applications.
